# Empowering recovery: the role of self-management in motor-functional neurological disorder

**DOI:** 10.3389/fneur.2026.1967513

**Published:** 2026-10-05

**Authors:** Sara Issak, Richard Antony Kanaan, Glenn Nielsen

**Affiliations:** 1 Department of Physiotherapy, Melbourne School of Health Sciences, The University of Melbourne, Parkville, VIC, Australia; 2 Department of Physiotherapy, Epworth Healthcare, Camberwell, Camberwell, VIC, Australia; 3 Department of Psychiatry, University of Melbourne, Austin Health, Heidelberg, VIC, Australia; 4 Neurosciences Research Centre, Molecular & Clinical Sciences Research Institute, City St George’s University of London, London, United Kingdom

**Keywords:** functional neurological disorder (FND), motor symptoms, recovery, rehabilitation, self-management

## Abstract

A Self-management refers to patients’ independent use of knowledge and skills to manage symptoms, maintain health, and respond to setbacks, both during and beyond active therapy. This mini-review summarises the current literature on self-management in motor-FND, including its key components, definitions, and role in supporting recovery during rehabilitation. FND-focused education, strategies to normalise movement, goal setting with graded activity, and the development of a relapse-prevention plan are described as components of self-management. Empirical evidence is limited but promising. Future studies need to examine and operationalise self-management strategies, including the optimal content, type, intensity and delivery. Self-management should empower individuals with motor-FND to become active agents in managing their condition.

## Introduction

Motor-Functional Neurological Disorder (motor-FND) is one of the two main subtypes of Functional Neurological Disorder (FND), characterised by motor disturbances such as tremor, weakness or gait disorder. For motor-FND, an interdisciplinary, biopsychosocial rehabilitation approach, integrating medical, psychological, and allied health interventions, is widely regarded as best practice. Studies often highlight the need for patients to use self-management (SM) strategies during and beyond the initial interventions (1–4). This can raise several challenges. While self-management is widely recognised as a key component of care in chronic neurological conditions, its operationalisation varies across the literature. It encompasses multiple components that require tailoring to the heterogeneous symptoms and needs of individuals. Implementation of SM draws on a range of clinical skills within a biopsychosocial framework, including effective communication, supporting patients’ understanding, and facilitating behaviour change. This complex range of skills and approaches makes it challenging for clinicians to translate SM principles into routine practice in motor-FND, highlighting the need for clearer clinical guidance, practical training and support, to enable effective and consistent implementation.

This paper provides an overview of the current literature on SM in motor-FND, exploring its key components, definitions, and role in supporting recovery during rehabilitation.

## What is self-management?

In this review, SM refers to the patient’s independent use of knowledge and skills to manage symptoms, maintain health, and respond to setbacks with a degree of autonomy.

Self-management interventions for chronic conditions aim to support informed decision-making, adoption of adaptive perspectives and skills, new health-related behaviours, and emotional stability (5). Rather than simply educating or promoting treatment adherence, they focus on facilitating behaviour change (6, 7) and encouraging people to play an active role in managing their condition (8). Stroke rehabilitation research has done much of the groundwork here, breaking down self-management into *problem-solving, goal setting, decision-making, self-monitoring, coping strategies,* and *approaches to sustain or enhance physical and psychological functioning* (Figure 1) (7). Self-management in stroke rehabilitation is delivered by health professionals, individually or in groups, via face-to-face or online modes, with written information provided, often involving multiple sessions (9).

**Figure 1:**
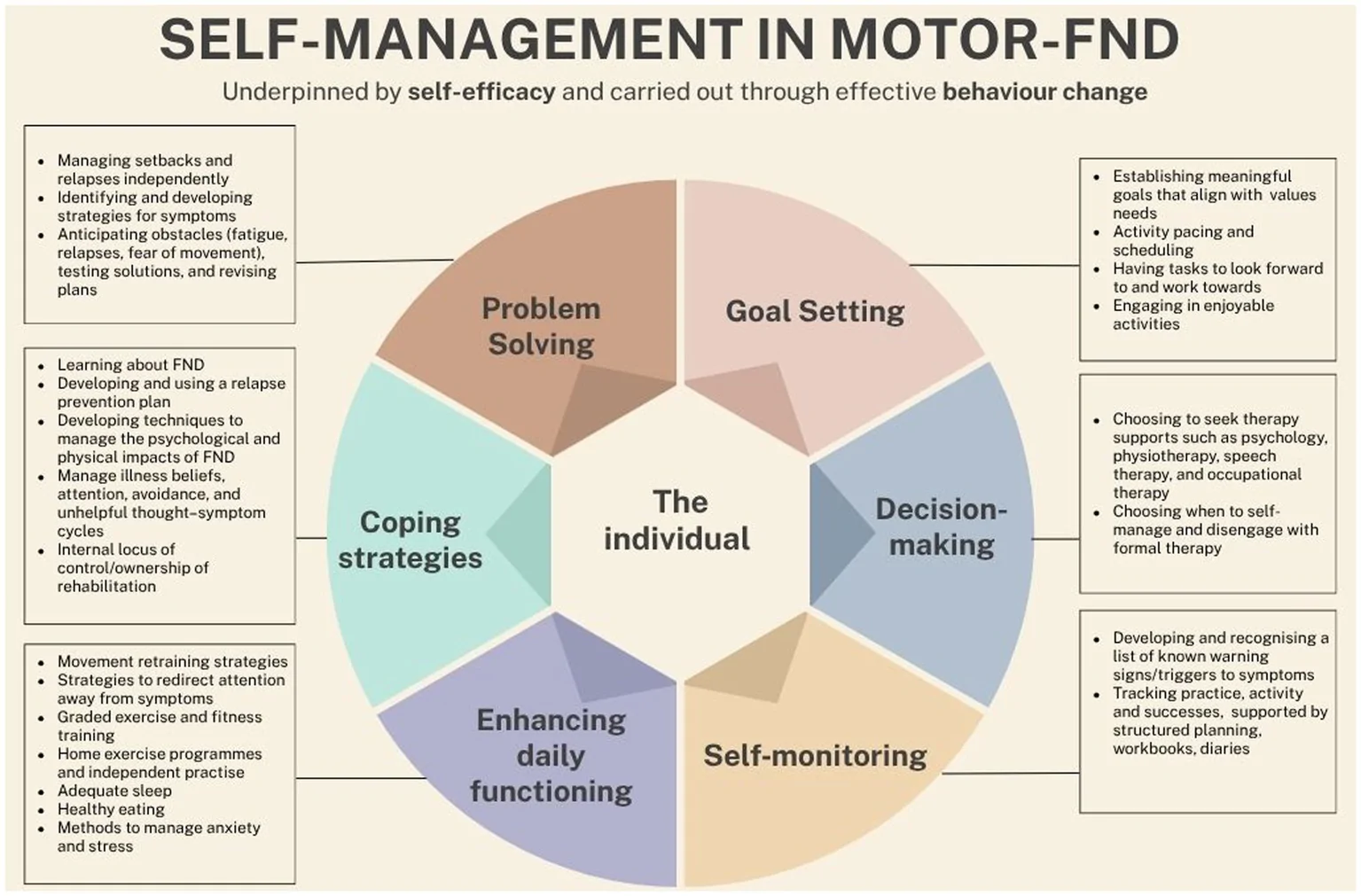
Conceptual summary of Self-Management in Motor-FND. Adopting the components of self-management from stroke rehabilitation, this figure summarises their application to self-management in motor-FND, underpinned by self-efficacy and health-related behaviour change.

## What is the evidence for self-management in rehabilitation?

Evidence from the broader rehabilitation literature supports self-management as an important component of long-term care for chronic health conditions. Systematic reviews across stroke, musculoskeletal and other chronic disease populations have demonstrated small but consistent improvements in functional outcomes, quality of life, health behaviours and self-efficacy following self-management interventions (10–12). Although effect sizes are generally modest and intervention approaches vary considerably, self-management programmes that incorporate goal setting, action planning, self-monitoring, problem-solving, education and social support appear to be associated with the greatest benefits (11, 13–15). These components may support rehabilitation by strengthening patients’ confidence in their ability to manage symptoms, activity and daily tasks. Accordingly, self-efficacy has been identified as a key mediator of treatment effects. Measured improvements in self-efficacy are associated with enhanced participation, functional independence and quality of life. These findings suggest that SM may be an important mechanism for maintaining rehabilitation gains beyond formal treatment and support the relevance of SM approaches in motor-FND rehabilitation.

## Self-management strategies in motor-FND

Based on the understanding of SM components in stroke rehabilitation, the motor-FND field can draw valuable parallels to optimise its own SM interventions. Self-management in the motor-FND literature is described as a discrete intervention (such as problem-solving for symptom setbacks) and the integration and application to other interventions (such as movement retraining). It has been described as “the patient taking ownership of their rehabilitation” and “the development of an internal locus of control” with emphasis on patient empowerment and autonomy (3).

Components of self-management in motor-FND rehabilitation include FND-focused education, learning strategies to normalise motor control, collaborative goal setting, and the development of a relapse-prevention plan (2, 3, 16). In addition, self-managing co-existing health issues such as pain, fatigue, sleep disturbances and psychological health are described (17, 18).

### FND-focused education

Helping patients understand their FND is considered one of the most important and initial components in treatment (19, 20). The literature supports the view that making a clear, respectful diagnosis with a good explanation is part of the treatment, being directly therapeutic in some cases, and strongly improving engagement with further therapy (21, 22). Multiple papers describe FND-focused education as a component of SM in motor-FND, which ideally supplements the psychoeducation already given by the diagnostician (2, 3, 16). FND education provides an understanding of the condition, its basic mechanisms and treatment principles, and empowers the use of this knowledge therapeutically. For example, an understanding that excessive self-directed attention worsens symptoms can help patients use strategies that helpfully redirect attention.

The benefit of FND-focused self-help education was explored in a randomised controlled trial (23). One hundred and eighty-six adults with motor-FND were randomised to either usual care or usual care plus access to an unguided, educational self-help website. The website aimed to improve understanding and encourage patients to take an active role in treatment, based on a biopsychosocial model of motor-FND. Results indicated that there was no added benefit from the educational self-help on self-rated health or symptoms at either 3 or 6 months; however, satisfaction was high among users (86% recommended it). The authors concluded that an online educational resource and non-guided self-help may be a valuable stepped-care adjunct, but not an effective stand-alone treatment.

### Strategies to normalise motor control

Movement retraining with diverted attention is a crucial component of motor-FND treatment and an important foundation for self-management. While movement retraining is typically introduced and practised within physiotherapy, its self-management application occurs when patients independently use these strategies to manage symptoms and maintain function in daily life (2, 24). Movement retraining aims to provide the patient with an experience of normal movement and to avoid reinforcement of altered movement patterns. Successful movement retraining strategies can be prescribed as homework and practised between physiotherapy sessions to improve mobility and balance (16). For example, weight-shifting (side-to-side and forward and backward) allows the automatic activation of ankle muscles, which may help initiate a normal gait pattern. Successful movement retraining may empower the patient to have a sense of control over their symptoms, reduce fear and worry from symptoms, reduce avoidance, promote improved daily function and reduce the impact of relapses.

Self-management principles are incorporated into the application of movement retraining in several studies (3, 4, 25–31). The physio4FMD randomised controlled trial, which compared usual-care physiotherapy with specialist-FND physiotherapy, provided a detailed example of this approach. The specialist intervention used individualised sequential motor learning exercises with the aim of building symptom-free movement patterns. Workbooks were used to support SM by recording information such as how to apply useful movement strategies, to monitor change using outcome measures (e.g., walking speed or balance scores), and to record patient reflections on their treatment sessions. The trial found no between-group difference in physical functioning as assessed by the 36-item short form survey (primary outcome measure). However, compared to usual care, more participants in the specialist physiotherapy group rated their motor symptoms as improved, and this group had better scores on measures of mental health and self-efficacy.

### Collaborative goal setting

Goal setting is a collaborative process whereby the clinician and patient develop meaningful activity-based goals to direct treatment. Graded goal setting describes the process of establishing small, achievable components of that goal, with plans to progress over time. It is a key element of SM, as described in separate consensus recommendations from physiotherapy, occupational therapy and speech therapy (2–4). There is an emphasis that rehabilitation goals should be formulated around functionally relevant and meaningful activities (4), and applied to everyday life roles and activities of daily living (3).

### Self-management plan development

Co-developing a written self-management plan is described as a specific component of treatment in consensus recommendations (2–4). These plans typically include information about how to manage setbacks and periods of symptom exacerbation. Nicholson et al. (3) describe it as an essential part of therapy, completed collaboratively at the conclusion of treatment. Recommendations include documenting: (1) the patient’s understanding of FND, (2) any triggers and eases to symptoms, (3) the most helpful management strategies, (4) any unhelpful coping methods that were preventing improvement, (5) an action plan for when function declines, or symptoms occur, and (6) ongoing goals. Several tools can support the development of a self-management plan, including a rehabilitation diary or workbook to record important strategies that normalise movement, keep track of goals, record achievements and exercise or activity plans (2). In addition, patients are informed that symptom setbacks are common in the post-treatment period, but the self-management plan can support them in getting back on track (32).

### Management of co-occurring health issues

Co-occurring pain, fatigue, sleep problems and mental health issues are common in motor-FND (17, 18). Developing self-management skills for co-occurring health problems is equally important, as they may interact with and amplify motor-FND symptoms. Self-management strategies may include pacing and graded activity to avoid boom–bust cycles for pain and fatigue (3), consistent sleep routines and hygiene protocols (16, 33), and strategies to reduce anxiety and stress, such as progressive muscle relaxation, breathing techniques and thought reframing (3).

### Health-related behaviour change

Behaviour change methods that support self-management should also be considered. Self-management requires individuals not only to acquire knowledge and skills, but also to adopt and sustain new ways of responding to symptoms, engaging in activity, and managing setbacks. Psychological constructs that support behaviour change include motivation and self-efficacy (defined as one’s belief about their capabilities to produce a certain level of performance) (6). Behaviour change theories may provide useful frameworks to implement SM strategies within clinical practice (34). While a detailed discussion of behaviour change theory is beyond the scope of this paper, approaches that enhance confidence, promote achievable goal attainment, support problem-solving and encourage active participation may be helpful.

Overall, across these domains, self-management can be conceptualised as a process that begins with clinician-supported learning and progresses towards independent implementation. Common underlying processes include developing an understanding of the condition, skill development, self-monitoring, problem-solving and the gradual development of self-efficacy. Figure 1 depicts a conceptual summary of SM strategies in the motor-FND field, anchored by the overarching principles of SM, adopted from the stroke literature.

## Evidence for self-management in motor-FND

Few studies have specifically examined self-management interventions for motor-FND, and the available evidence largely considers SM as part of broader rehabilitation programmes. One study that provides useful insights compared telehealth support with a self-management programme (35). Sixty-four patients with motor-FND underwent a 5-day, in-person rehabilitation program followed by either a self-management (32 patients) or a telehealth program (32 patients). The study examined outcomes on motor and non-motor symptoms such as fatigue and pain, quality of life, perception of change, gait, and postural control at 3 months. Results showed that in the telehealth group, improvements were greater than in the self-management group for motor symptoms (*p* < 0.001), physical fatigue (*p* = 0.028), and self-rated change perception (*p* = 0.043). Interestingly, the same research group examined the long-term outcome of telemedicine support, compared to SM support, and found that at 36-month follow-up, participants had sustained perceived improvements in the telehealth group, despite an absence of additional motor benefits (36). One explanation for this outcome, if we consider the telehealth arm of this study to have received ‘supported self-management’, is that SM is more than simply transferring responsibility from clinician to patient; it is a supported, collaborative process. However, this was a small, single-site study, and the results would need to be replicated in a larger sample.

Overall, these studies suggest that SM may work best as an adjunct to support rehabilitation, providing promising evidence; however, future research is required to clearly operationalise and systematically examine the components of SM to identify specific treatment mediators. Given the limited evidence for SM in motor FND, it may be helpful to consider what can be learned from the literature on functional dissociative seizures.

## Learnings from self-management in functional dissociative seizures

Self-management in functional dissociative seizures (FDS) incorporates a range of psychological and behavioural symptom-management strategies, with emphasis placed on improving patients’ ability to manage and respond to symptoms rather than solely reducing seizure frequency (37). Emphasis is given on patient empowerment towards independent symptom management, coping strategies, improved functional outcomes and quality of life. In FDS, these strategies are used both proactively (before FDS onset, during warning signs) and reactively (after FDS onset), with emphasis on proactive approaches (38).

A 5-session face-to-face or telehealth intervention titled ‘Re-PROGRAM’, developed to teach seizure management skills, lifestyle modification, and coping strategies, was found to be feasible with patient-reported improvements in seizure-control confidence, seizure intensity, and daily functioning (39), with emphasis on enabling SM. The CODES trial, a large multicentre randomised controlled study, evaluated cognitive behavioural therapy (CBT) plus standardised medical care versus standard care alone for adults with FDS (40). While CBT did not significantly reduce monthly seizure frequency at 12 months compared to standard care alone, it led to clinically meaningful improvements in several secondary outcomes, including longer seizure-free periods, reduced somatic symptoms, better health-related quality of life, improved psychosocial functioning, and decreased psychological distress. A mediation analysis revealed that CBT for FDS improved avoidance behaviours, anxiety, and depression, which was linked to better daily functioning and better mental quality of life (37). These studies suggest that self-management in FDS enhances coping and quality of life rather than directly reducing seizure frequency.

Learnings from the evidence base for SM in FDS that may be applied to motor-FND include: (1) A focus beyond symptoms alone - in FDS seizure frequency improvements may be modest, but the emphasis is on improving functioning, mood and quality of life; (2) Building coping skills and education to promote problem-solving for set-backs; (3) Proactive as well as reactive strategies, which are especially important for paroxysmal motor symptoms, and can be considered within individualised action plans.

## Recommendations for implementing self-management

The interdisciplinary team shares the responsibility for introducing the concept of SM to people with motor-FND during treatment. Alongside the patient, they should develop and compile a self-management summary document that the patient can refer to after treatment is completed. Practically, this can commence at the beginning of treatment as a working document that all team members involved in care can contribute to, or at the end of treatment by dedicating at least one session to bringing together all relevant components of SM, with the patient.

The form and type of SM recommendations may vary among treating clinicians working with people with motor-FND; however, significant overlap and shared effectiveness are evident. Typically, physiotherapists may provide strategies for movement retraining and managing motor symptoms, pain, fatigue, as well as activity recommendations and home exercise programmes (2). Self-management recommendations from occupational therapists include supporting task and occupational practice, whilst integrating SM strategies. This can include recommendations for a graded return to daily activities and usual occupations such as work or study, supported by activity pacing and fatigue management (3). Speech therapists provide SM tools through exercises and strategies to minimise the impact of speech, communication and swallow symptoms (4). Psychologists provide cognitive behavioural techniques to manage anxiety, fear, worry and symptom burden, challenge maladaptive beliefs, and unhelpful illness behaviours (40, 41). In reality, disciplinary roles are not so clearly divided. Psychological tools may reduce the impact of physical symptoms, and movement retraining strategies may reduce psychological and emotional difficulties. This blurring of boundaries, and the use of shared approaches across disciplines, is characteristic of a transdisciplinary approach to treatment. This contrasts with an interdisciplinary approach, which integrates care through goal setting and regular, active collaboration, while each team member remains within their own disciplinary scope (42). The transdisciplinary approach is grounded in person-centred care, prioritising the person’s needs over discipline-specific boundaries.

## Co-designing self-management resources

Existing literature on co-design and patient and public involvement, in healthcare more broadly, has emphasised the meaningful involvement and embedding of lived experience from consumers early on in projects (43). Within FND specifically, a co-produced symptom self-management resource was developed, through a quality improvement project at a neuropsychiatry service based in the United Kingdom, resulting in a refined symptom self-management booklet, a symptom recording template, and educational resources, using structured input from patients (44). Across this body of work, people with lived experience are positioned not merely as end-users providing feedback on a finished product, but as active contributors shaping content, format, and delivery. Future work is needed to co-develop SM resources in motor-FND that work for those with the condition, improving the relevance, acceptability, and uptake of self-management resources.

## Conclusion and future directions

Self-management in motor-FND can be understood as the application of strategies to manage the impact of symptoms during and beyond active treatment. It aims to help people understand their condition, develop coping strategies, and carry these changes into daily life with growing confidence and independence. Self-management in motor-FND is a core therapeutic priority, empowering patients to become active agents in managing their condition.

Evidence from the broader rehabilitation literature provides a strong rationale for incorporating self-management principles into motor-FND treatment. Although the FND-specific evidence base remains limited, existing studies, clinical models and expert consensus support the view that self-management is likely to be an important part of effective rehabilitation.

Further research is needed to determine which self-management approaches are most effective for people with motor-FND, how they should be tailored to individual needs, and how clinicians can best support patients in maintaining improvements over time. Importantly, consultation with patients and those with lived experience is needed to co-design SM resources and templates. Studies are also needed to examine how SM can be effectively implemented and evaluated in clinical settings, with a focus on supporting clinician training.
